# Invasions Slow Down or Collapse in the Presence of Reactive Boundaries

**DOI:** 10.1007/s11538-017-0326-x

**Published:** 2017-08-01

**Authors:** K. Minors, J. H. P. Dawes

**Affiliations:** Department of Mathematical Sciences, Centre for Networks and Collective Behaviour, University of Bath, Claverton Down, Bath, BA2 7AY UK

**Keywords:** 2D FKPP equation, Mixed boundary conditions, Strip invasion, Population collapse, Critical patch size

## Abstract

Motivated by the propagation of thin bacterial films around planar obstacles, this paper considers the dynamics of travelling wave solutions to the Fisher–KPP equation $u_t = u(1-u) + u_{xx} + u_{yy}$ in a planar strip $-\infty< x < \infty$, $0 \le y \le L$. We examine the propagation of fronts in the presence of a mixed boundary condition (also referred to as a ‘partially absorbing’ or ‘reactive’ boundary) $u_y = \alpha u$, with $\alpha >0$, at $y=0$. The presence of boundary conditions of this kind leads to the development of front solutions that propagate in *x* but contain transverse structure in *y*. Motivated by the observation that the speed of propagation in the Fisher–KPP equation is determined (for exponentially decaying initial conditions) by the behaviour at the leading edge, we analyse the linearised Fisher–KPP equation in order to estimate the speed of the stable travelling front, a function of the width *L* and the imposed boundary conditions. For wide strips the speed estimate based on the linearised equation agrees well with the results of numerical simulations. For narrow channels numerical simulations indicate that the stable front propagates more slowly, and for sufficiently small *L* or sufficiently large $\alpha$ the front speed falls to zero and the front collapses. The reason for the collapse is the non-existence, far behind the front, of a stable positive equilibrium solution *u*(*x*, *y*). While existence of these equilibrium states can be demonstrated via phase plane arguments, the investigation of stability is similar to calculations of critical patch sizes carried out in similar ecological models.

## Introduction

Many biological situations can be viewed as invasions of one population into another; either a new genetic trait or species out-competing an existing one for resources, or consuming a preexisting resource distributed over a spatial domain. For such situations a number of simple mathematical models have been proposed in order to quantitatively capture spatial aspects of the population dynamics and the propagation of the new species (Volpert et al. 1994; Shigesada and Kawasaki 1997; Murray 2002; Volpert and Petrovskii 2009; Lewis et al. 2016). The simplest situation considers the density of the invading population to be described by a single scalar variable $u(\mathbf {x},t)$ that is a function of space and time. Further common simplifying assumptions are that the background state $u=0$ is homogeneous, and locally unstable, i.e. that a small positive density of invaders *u* will grow over time. It is also plausible from biological considerations to argue that solutions should always remain bounded and hence the density *u* will saturate to a constant value at long times; without loss of generality we take this to be the state $u=1$.

The simplest model of this kind describing propagation into a state where *u* is initially close to zero and then rises to saturate at $u=1$ is the well-known Fisher–Kolmogorov–Petrovskii–Piskunov (FKPP) equation (Fisher 1937; Kolmogorov et al. 1937) which, in two spatial dimensions, takes the form

1 $$\begin{aligned} u_t = u(1-u) + u_{xx} + u_{yy}, \end{aligned}$$

where $u=u(\mathbf {x},t)$ and $\mathbf {x}\equiv (x,y) \in \Omega _L:= (-\infty ,\infty ) \times [0,L] \subseteq \mathbb {R}^2$. Our interest in this paper is in solutions in the form of ‘fronts’, i.e. solutions that for every fixed *y* and *t* are monotonically decreasing in *x* and which satisfy $u(-\infty ,y,t)=1$ and $u(\infty ,y,t)=0$. Instead of the domain $\Omega _L$ we occasionally consider the half-space $\Omega :=(-\infty ,\infty ) \times [0,\infty )$. Fronts therefore propagate from left to right along the *x*-axis. Travelling wave solutions are those for which *u* takes the form $u(x,y,t)=\tilde{u}(x-ct,y)$ where *c* is the wave speed.

The corresponding problem in one spatial dimension, looking for *y*-independent solutions to (1) is discussed in detail by Volpert et al. (1994). For the case considered in this paper (the monostable case in which the form of the nonlinear term implies that the state $u=0$ is unstable and $u=1$ is stable), phase plane analysis shows that travelling waves cannot exist for wave speeds $c < 2$, but do exist for all speeds $c \ge 2$ (Harris 1999).

Only at isolated specific values of the wave speed is it possible to find an exact analytic travelling wave solution to (1), one example is the exact solution

$$\begin{aligned} u(x,t) = \left\{ \frac{1}{2} \tanh \left( \frac{x-5t/\sqrt{6}}{2\sqrt{6}} \right) + \frac{1}{2} \right\} ^2 , \end{aligned}$$

deduced by Ablowitz and Zeppetella (1979) which travels at a speed $5/\sqrt{6}\approx 2.041$ and which is an exact solution to (1) when *u* is taken to be independent of *y*. Murray (2002), citing earlier work by Canosa (1973), outlines an asymptotic approach to approximation of the form of travelling waves, taking the reciprocal of the wave speed 1 / *c* to be a small parameter.

Stability issues have also been investigated in detail by many authors. In one spatial dimension it is well established that the solution to the initial value problem with exponentially decaying initial conditions (for example compactly supported initial conditions) will converge to a travelling wave solution that propagates with the minimum allowable speed $c=2$. This was proved by Larson (1978) building on work of McKean (1975), and numerically verified by Manoranjan and Mitchell (1983). Initial conditions that decay more slowly converge to travelling waves that propagate faster, see for example Hamel and Roques (2010), Sherratt (1998) and the references therein.

Our interest in this paper is to consider extensions of the 1D Fisher–KPP problem to two-dimensional spatial domains. Clearly one-dimensional solutions are also exact solutions of the 2D Eq. (1) when it is posed either in an infinite spatial domain $(x,y) \in \mathbb {R}^2$, or in the domains $\Omega$ or $\Omega _L$ defined above, with a Neumann boundary condition $\mathbf {n}\cdot \nabla u =0$ imposed everywhere.

In the case in which the domain is $\mathbb {R}^2$, families of exotic travelling wave solutions have been studied through analysis of the linearised problem by Brazhnik and Tyson (2000), see also Showalter (1995). In the case of the strip $\Omega _L$, the evolution of fronts for the Fisher–KPP equation becomes connected to ecological questions of the critical patch size for the maintenance of the population since a necessary condition for the propagation of a travelling wave solution is that the population should be able to maintain itself at an equilibrium density in the region behind the travelling wave. This is an important question in the case we study here since reproduction and dispersal in a finite domain compete with extinction, or at least a reduced reproduction rate, outside the finite domain.

The effect of a finite domain size on population dynamical models in general has been studied by many authors, stretching back to Skellam (1951). It is well known that, for simple ecological models such as the 1D version of (1), say for *u*(*y*, *t*) independent of *x*, the imposition of Dirichlet boundary conditions $u(0,t)=u(L,t)=0$ implies that a population cannot persist if the domain length *L* is less than a critical length $L_c:=\pi$ (Kierstead and Slobodkin 1953; Ludwig et al. 1979).

In more general cases in which the environment for the population growth and dispersal changes unfavourably along the boundaries, rather than taking Dirichlet boundary conditions $u=0$ at $y=0$ and $y=L$, more realistic boundary conditions are the mixed-type (or ‘reactive’, or ‘partially absorbing’) boundary conditions $u_y = \alpha u$ at $y=0$ and $u_y=-\beta u$ at $y=L$, where $\alpha ,\beta \ge 0$. This corresponds to situations where the Fisher–KPP equation is motivated as a continuum description arising from a microscopic model in which, when an individual crosses the boundary there is a positive probability of absorption onto the boundary rather than reflection back into the interior of the domain (Erban and Chapman 2007). Such partially absorbing behaviour has been studied experimentally observing the propagation of bacteriophages through populations of *E. coli*. Experimental observations indicated that the propagation fronts of the invading bacteriophage population developed a curved profile near the boundaries of the available bacterial population. This phenomenon could not be reproduced by assuming a no-flux boundary condition (Möbius et al. 2015), which provided the original motivation for the consideration of the partially absorbing boundary conditions that we use here.

The structure of this paper is as follows. In Sect. 2 we examine the linearised problem at the leading edge of a front in the Fisher–KPP equation. We solve the linear problem and hence deduce the speed *c* of the front in terms of the parameters that determine the reactive boundary condition. As a result we are able to estimate analytically how the presence of the reactive boundaries cause the travelling fronts to slow down in wide strips.

In Sect. 3 we discuss the form of the fully nonlinear travelling front far behind the leading edge. This also turns out to be analytically tractable. It also explains the shape of contours of constant population density when plotted in the (*x*, *y*) plane to describe or detect the travelling front, for example experimentally.

As the strip becomes narrower, the population density behind a stable propagating front is observed numerically to decrease. This motivates, in Sect. 3.2 an investigation of whether there is a critical width below which the strip is unable to sustain a positive population behind the front. This is indeed the case. In sufficiently narrow strips, when $\alpha$ and $\beta$ are sufficiently large, the population density falls to zero behind the front and the solution collapses to zero everywhere: there appears to be no stable travelling wave in this regime.

Section 4 summarises these results, compares them with previous results for critical patch sizes, and outlines directions for future work.

## The Speed of the Front

The FKPP equation is a basic model for the propagation of a stable state $u=1$ into an unstable state $u=0$. The speed of propagation is expected to be controlled by the dynamics at the leading edge of the front since this is a pulled front (Saarloos 2003). We consider the FKPP equation posed in a strip $(x,y) \in \mathbb {R}\times [0,L]$. We solve the initial value problem always considering compactly supported initial conditions. We therefore expect (but have not proved) that solutions exist and are unique, and converge to the unique solution that travels with the slowest available speed.

Our interest is in the transverse structure (i.e. the variation of *u*(*x*, *y*, *t*) in the *y* direction) at the leading edge of the front. If the boundary conditions at $y=0$ and $y=L$ are zero flux (Neumann) conditions $u_y=0$ then we would expect that there is no transverse variation and the problem is one-dimensional: solutions converge to travelling wavefronts that are independent of *y*. It is not *a priori* clear that this should be the case with Neumann boundary conditions, but numerical solutions provide evidence that this is indeed the case.

Imposing reactive (or mixed, or Robin) boundary conditions of the form

2 $$\begin{aligned} u_y=\alpha u \qquad \text {at} \qquad y=0, \qquad \text {and} \qquad u_y=-\beta u \qquad \text {at} \qquad y=L, \end{aligned}$$

does, however, generate transverse structure as we now show. In the region $x \gg 0$ the amplitude *u* is small so that the FKPP equation can be well approximated by its linearisation

3 $$\begin{aligned} u_t=u_{xx} + u_{yy} + u. \end{aligned}$$

**Fig. 1:**
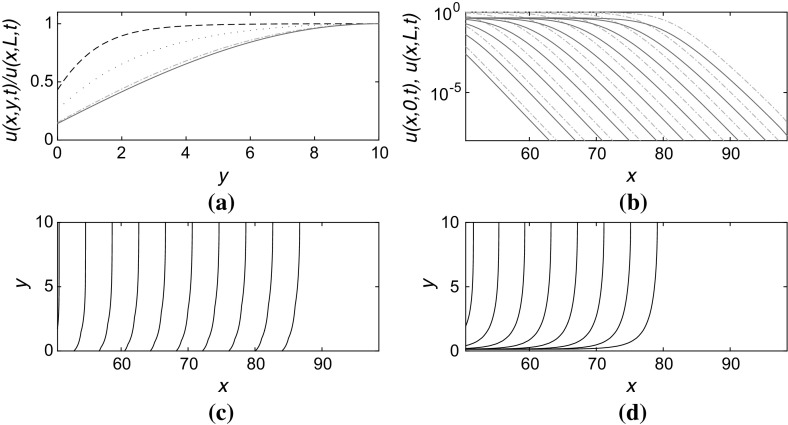
(Color figure online) Numerical solution to (1) in a channel $0 \le x \le 100$, $0 \le y \le L$ with boundary conditions $u_y=\alpha u$ at $y=0$ and $u_y=0$ at $y=L$, taking $\alpha =1.0$ and $L=10$. **a** Transverse structure of the front in the *y*-direction at $x=70,80,90$ (*dashed*, *dotted*, and *dash-dotted lines*, respectively) at the timepoints corresponding to the rightmost contours in **c** and **d**. *Solid line* indicates the (*rescaled*) profile at the leading edge, agreeing with the *dash-dotted line* at $x=90$ which is well ahead of the front at this moment in time. *Dotted line* for $x=80$ and *dashed line* for $x=70$ are at, and behind, the front, respectively, showing the form of the front in the fully nonlinear region. **b** Propagation of the front from *left* to *right* at time increments $\Delta t=2.0$ showing *u*(*x*, 0, *t*) (*solid blue lines*) and *u*(*x*, *L*, *t*) (*dash-dotted red lines*). The linear-log *scale* indicates the exponential decay of the front. **c** Contours of constant $u=10^{-2}$ at times separated by $\Delta t=2.0$. **d** Contours as in **c** but for $u=0.5$

We substitute the separable ansatz $u=\mathrm {e}^{-(x-ct)}v(y)$ suitable for a constant coefficient linear PDE, and assuming that the leading edge of a stable front will decay exponentially in the *x* direction with exponent $-1$ as in the case in one dimension. This decay rate of the leading edge is confirmed by numerical investigations, as shown for example in Fig. 1b.

With this assumption, the speed *c* of propagation of the front is determined from the resulting eigenvalue equation for *v*(*y*):

4 $$\begin{aligned} v_{yy} + (2-c)v = 0, \end{aligned}$$

together with boundary conditions of the form (2). We now consider three cases in turn; in every case we impose the boundary condition $v_y=\alpha v$ at $y=0$, but we consider in turn imposing either a no-flux boundary condition $u_y=0$ at $y=L$, or a reactive boundary condition $u_y=-\beta u$ at $y=L$, or in the third case we consider (4) to be posed in the half-space $y \ge 0$ with only a single boundary condition required (at $y=0$).

### One Partially Absorbing Boundary and One No-Flux Boundary

For the case in which the boundary at $y=0$ is partially absorbing: $v_y = \alpha v$, and for which *v* satisfies a no-flux boundary condition at $y=L$, i.e. $v_y=0$ at $y=L$, we would intuitively expect that the speed *c* is reduced below the value 2 which would correspond to the 1D FKPP front speed (i.e. the case $\alpha =0$. Given the form of (4) it is convenient to introduce the new variable $p:=\sqrt{2-c})$. Then the solution for *v* takes the form

5 $$\begin{aligned} v(y)=A \left( \cos py + \frac{\alpha }{p}\sin py \right) , \end{aligned}$$

where *A* is an undetermined multiplicative constant, and *p* is the smallest positive solution of the equation

6 $$\begin{aligned} \tan pL = \alpha /p. \end{aligned}$$

Although (6) has a countable collection of solutions for *p* (eigenvalues), the physically relevant one is the leading eigenvalue (the smallest positive value of *p*) since this leads to the linear eigenmode with the largest positive growth rate and therefore this mode will dominate the solution for the leading edge of the front. We note that the corresponding eigenfunction *v*(*y*) is monotonically increasing, which agrees with our intuition about the biological interpretation of the reactive boundary condition being to reduce the population density in the vicinity of the boundary.

The form (5) provides a very good approximation to the shape of the leading edge of the front, as shown in Fig. 1. Figure 1a shows the transverse (i.e. *y*-direction) structure of the front at three positions $x=70,80,90$ when the front has reached the rightmost location shown in each of Fig. 1b–d. The solid line in Fig. 1a is the theoretical form of the leading edge of the front; the dash-dotted line is very close to it since at this moment in time the leading edge of the front is at the position $x=90$ at which the (rescaled) profile *u*(90, *y*, *t*) / *u*(90, *L*, *t*) is plotted. The dotted line is a slice through the domain at $x=80$ which is through the middle of the front, departing from the profile computed from the leading edge. The dashed line, at $x=70$ is well behind the front and shows the influence of the partially absorbing boundary condition at $y=0$ where the population density is significantly reduced compared to its value at $y=L$ where a no-flux boundary condition is imposed.

Figure 1 and the similar subsequent figures were obtained from straightforward numerical simulations (spatial finite differences and explicit timestepping) and initial conditions that decayed exponentially in space. Implementation of the partially absorbing boundary conditions are straightforward in this case. For all results shown, the spatial and temporal resolution was sufficiently high that solutions were well-converged. Part (b) of the figure plots the shape of the front *u*(*x*, *y*, *t*) at the two locations $y=0$ and $y=L$, spaced by time intervals $\Delta t=2.0$ showing the propagation into $x>0$ at a constant velocity. At each timepoint and each point *x*, the value *u*(*x*, *L*, *t*) (shown by the red dash-dotted line) is larger than *u*(*x*, 0, *t*) (shown by the blue solid line). Figure 1c, d shows contours of constant *u* as time increases. In Fig. 1c, we observe that the front remains parallel to the boundary $y=L$ where the no-flux condition is applied, but bends close to the partially absorbing boundary at $y=0$. For the larger value for contours shown in Fig. 1d the contours do not extend all the way down to the boundary at $y=0$ since the values of *u* there always remain below a value $u \approx 0.4423$ in this case. This aspect is discussed further in Sect. 3.

**Fig. 2:**
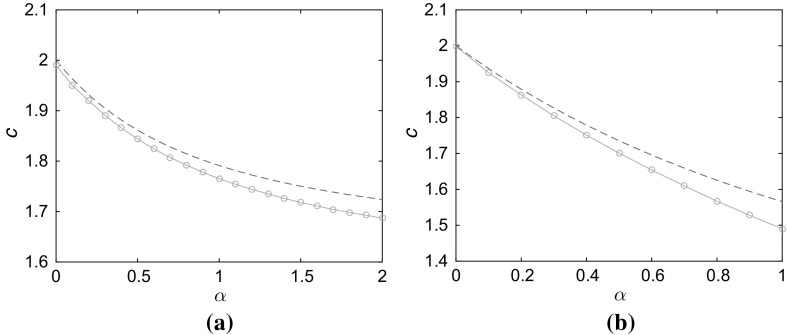
(Color figure online) Front propagation speed *c* as a function of $\alpha$, setting $\beta =0$ and taking **a** $L=2.5$ and **b** $L=1.5$. *Solid curves* indicate speeds computed numerically; *dashed lines* indicate theoretical predictions using (6)

Equation (6) can therefore be viewed as implicitly determining the front propagation speed *c* as a function of $\alpha$. In particular it is interesting to note that (6) predicts that $c=0$ when $p=\sqrt{2}$, i.e. on the line

7 $$\begin{aligned} \alpha =\sqrt{2} \tan (L\sqrt{2}). \end{aligned}$$

This result has two implications. Firstly, for any fixed positive $\alpha$ there is a minimum strip width $L_m(\alpha )$ on which $c=0$ and therefore below which the front cannot advance along the strip. Secondly, considering variations in *L*, there are two regimes: if

8 $$\begin{aligned} L > L_m^* := \frac{\pi }{2\sqrt{2}} \approx 1.11, \end{aligned}$$

then this calculation predicts that the front will always propagate forwards, for any value of $\alpha$ however large. But for $L<L_m^*$ from this linearised theory at the leading edge we would predict that the front would propagate forwards for sufficiently small $\alpha$ and would propagate backwards for sufficiently large $\alpha$, as long as a travelling wave solution exists for these parameter values. Note that $\lim _{\alpha \rightarrow \infty } L_m(\alpha ) = L_m^*$.

Figure 2 compares the results of the theoretical calculation at the leading edge (6) with speeds *c* estimated directly from numerical simulation. The front propagates significantly more slowly as $\alpha$ increases or when the strip becomes narrower, although for this range of parameter values the front always propagates forwards. It is interesting to note that, while the agreement is better for large strip widths *L*, the numerically computed value is always below the theoretical estimate. This is in contrast to the 1D FKPP problem for which numerical solutions (with compactly support initial conditions) produce stable fronts that travel at the lowest possible speed.

### Two Partially Absorbing Boundaries

The solution of (4) in the case of a mixed boundary conditions $v_y=\alpha v$ at $y=0$ and $v_y=-\beta v$ at $y=L$ (with $\alpha$, $\beta >0$) is similarly straightforward: the solution for *v*(*y*) still takes the form (5) but now the parameter *p* is the smallest positive solution of the equation

9 $$\begin{aligned} \alpha + \beta = \left( p - \frac{\alpha \beta }{p}\right) \tan pL. \end{aligned}$$

For fixed $\beta >0$ the line $L_0(\alpha )$ in the $(L,\alpha )$ plane along which the speed *c* of the front is predicted to fall to zero is given by setting $p=\sqrt{2}$ in (9) which gives

10 $$\begin{aligned} \alpha = \frac{2\tan (L\sqrt{2}) - \beta \sqrt{2}}{\sqrt{2} + \beta \tan (L\sqrt{2})}. \end{aligned}$$

Thus the critical strip width $L_m^*(\beta )$, below which the leading edge calculation indicates that the front speed could become zero for a sufficiently large value of $\alpha$, is given by

$$\begin{aligned} L_m^*(\beta ) = \frac{1}{\sqrt{2}} \left[ \pi + \tan ^{-1}_P \left( -\frac{\sqrt{2}}{\beta } \right) \right] , \end{aligned}$$

where $\tan ^{-1}_P$ denotes the principal value of the inverse tangent function.

**Fig. 3:**
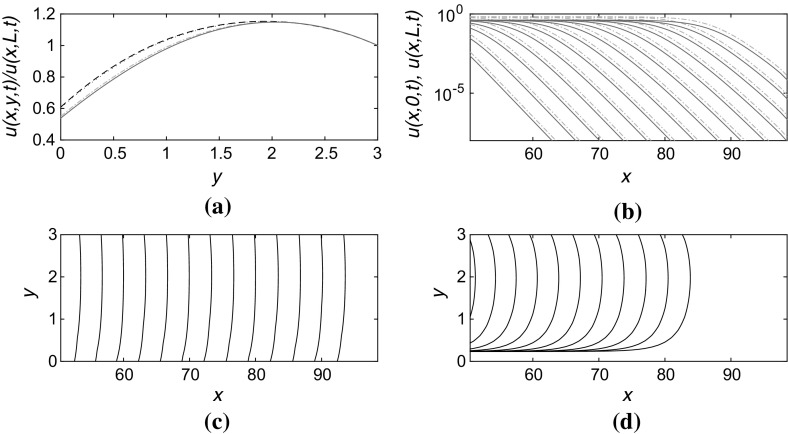
(Color figure online) Numerical solution to (1) in a channel $0 \le x \le 100$, $0 \le y \le L$ with boundary conditions $u_y=\alpha u$ at $y=0$ and $u_y=-\beta u$ at $y=L$, taking $\alpha =1.0$, $\beta =0.3$, and $L=3$. **a** Transverse structure of the front in the *y*-direction at $x=70,80,90$ (*dashed*, *dotted*, and *dash-dotted lines*, respectively) at the timepoint corresponding to the rightmost contours in **c** and **d**. The *solid line* is the theoretical prediction of the shape of the leading edge of the front. **b** Propagation of the front from *left* to *right* at time increments $\Delta t=2.0$ showing *u*(*x*, 0, *t*) (*solid blue lines*) and *u*(*x*, *L*, *t*) (*dash-dotted red lines*). The linear-log *scale* indicates the exponential decay of the front. **c** Contours of constant $u=10^{-2}$ at times separated by $\Delta t=2.0$. **d** Contours as in **c** but for $u=0.5$

**Fig. 4:**
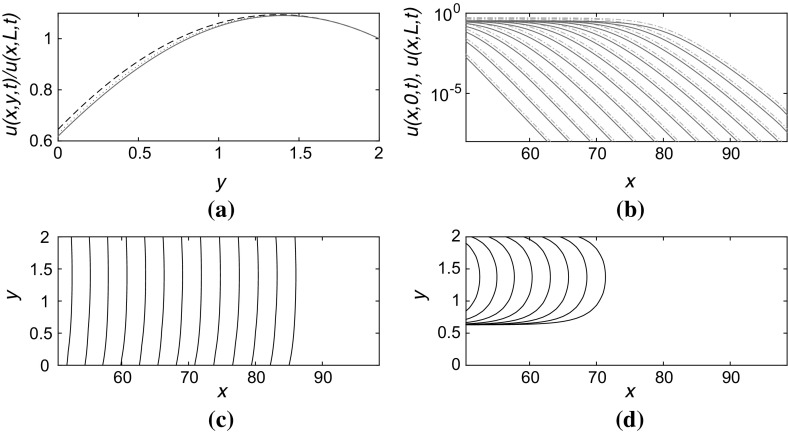
(Color figure online) Numerical solution to (1) in a channel $0 \le x \le 100$, $0 \le y \le L$ with boundary conditions $u_y=\alpha u$ at $y=0$ and $u_y=-\beta u$ at $y=L$, taking $\alpha =1.0$, $\beta =0.3$, and $L=2$. For details of plots, refer to the caption to Fig. 3. **a** Transverse structure of the front in the *y*-direction at $x=70,80,90$, respectively at the timepoint corresponding to the rightmost contours in **c** and **d**. **b** Propagation of the front from *left* to *right* at time increments $\Delta t=2.0$ showing *u*(*x*, 0, *t*) (*solid blue lines*) and *u*(*x*, *L*, *t*) (*dash-dotted red lines*). The linear-log *scale* indicates the exponential decay of the front. **c** Contours of constant $u=10^{-2}$ at times separated by $\Delta t=2.0$. **d** Contours as in **c** but for $u=0.5$

Figures 3 and 4 illustrate the propagation of fronts in narrow channels, with widths $L=3.0$ and $L=2.0$, respectively. The slowdown in the front propagation is clear from comparisons between parts (c) and (d) in each figure, while the linear approximation appears still to agree well with the numerically computed solution ahead of the front where $u \ll 1$. It is also interesting to note that the shape of the contours of constant *u* have a very different appearance depending on the level at which they are plotted. Figures 3d and 4d show that if the contour value is high, the contour may not extend to anywhere near the partially absorbing boundary: the front more resembles a blob that propagates through the centre of the channel and does not appear to touch the boundaries at all. This is potentially important for observing experimental results where the imaging technique may similarly not allow direct observation of the shape of the front at low values of *u* and hence might not follow the front shape all the way down to the boundary itself.

For completeness, we note that if $\alpha =\beta$ then this calculation predicts that a front can propagate through the domain only if the domain size *L* satisfies

11 $$\begin{aligned} L > {\frac{1}{\sqrt{2}} \tan ^{-1}} \left( \frac{2\sqrt{2}\alpha }{2-\alpha ^2} \right) . \end{aligned}$$

### Solution in the Half-Space $y \ge 0$

Now we turn to the case of (4) subject to only one boundary condition, $v_y=\alpha v$ at $y=0$. Far away from $y=0$ we would expect that the front solution resembles that for the 1D problem, and travels with speed $c=2$. Substituting the solution ansatz $u(x,y,t)=u_0 \mathrm {e}^{-(x-2t)}f(y)$ into (3) we require that $f_{yy}=0$, and $f'(0)=\alpha f(0)$ which implies $f(y)=f_0(1+\alpha y)$ with $f_0$ an undetermined constant. Hence in the half-space $y \ge 0$ the front always travels with speed $c=2$ independent of the value of the parameter $\alpha$.

The solution

12 $$\begin{aligned} u(x,y,t)=u_0 \mathrm {e}^{-(x-2t)} (1+\alpha y), \end{aligned}$$

to (3) allows us to examine the shape of level sets $u=const$ near $y=0$, far ahead of the front. Setting $u=u_0$ and $t=0$ without loss of generality, we find that the level set $u=u_0$ is described by the curve $x=\log (1+\alpha y)$ which has two features of note: even in the region $y \gg 1$ away from the boundary, the level set curves do not become asymptotically parallel to the *y*-axis, and also, expanding the solution for small *x* and *y* near the $y=0$ boundary we see that the level set takes the form $y=\frac{1}{\alpha }x$ here, so that level set curves meet the *x*-axis at an angle $\theta$ determined by $\alpha$ through the relation $\tan \theta =1/\alpha$.

This behaviour near the boundary, with the level sets making the angle $\tan ^{-1}(1/\alpha )$ with the *x*-axis, arises in the both the problems presented above for solutions in a strip $0 \le y \le L$; this behaviour is determined by the partially absorbing boundary condition alone and does not depend, as one might intuitively hope, on the choice of ‘far-field’ boundary condition.

## Behind the Front

Since there is no closed-form explicit solution even for the 1D FKPP equation, it is not possible to follow the evolution of the front solutions for the 2D problem into the fully nonlinear regime. However, it is possible to study the transverse (i.e. *y*-direction) structure of solutions far behind the front; we do this in Sect. 3.1 for each of the cases considered in the previous section. The simplest of these cases is the half-space $y \ge 0$, which we consider first, and for which an explicit solution profile can be obtained. We then consider solutions confined to a strip $0 \le y \le L$ for which the existence of unique monotonic fronts is clear geometrically, and for which an implicit form of the solution is available. In Sect. 3.2 we consider the stability of solutions, looking in particular at the regime where the population density behind the front becomes very low.

### Existence of Solutions

Consider solutions to (1) with boundary condition (2); far behind the front the solution *u*(*x*, *y*, *t*) becomes independent of *x* and *t* since in the 1D problem solutions tend to the constant 1. Hence we write $u(x,y,t) = w(y)$ where *w*(*y*) is required to solve the nonlinear ODE

13 $$\begin{aligned} w_{yy} = w (w-1), \end{aligned}$$

subject to the boundary conditions

$$\begin{aligned} w_y=\alpha w \ \mathrm {at} \ y=0, \ \ \mathrm {and} \ \ w \rightarrow 1 \ \mathrm {as} \ y \rightarrow \infty . \end{aligned}$$

Introducing the new variable $z=\mathrm{d}w/\mathrm{d}y$ allows us to write (13) as the two-dimensional Hamiltonian system

14 $$\begin{aligned} w_y = z = \frac{\partial H}{\partial z}, \qquad \mathrm {and} \qquad z_y = w(w-1)= - \frac{\partial H}{\partial w}, \end{aligned}$$

where the Hamiltonian *H* is given by

15 $$\begin{aligned} H = \frac{1}{2} z^2 + \left( \frac{1}{2} w^2 - \frac{1}{3}w^3 \right) , \end{aligned}$$

and the Jacobian matrix at a point (*w*, *z*) in phase space is

16 $$\begin{aligned} J_{(w,z)} = \left( \begin{array}{cc} 0 &{}\quad 1 \\ 2w-1 &{}\quad 0 \end{array}\right) . \end{aligned}$$

**Fig. 5:**
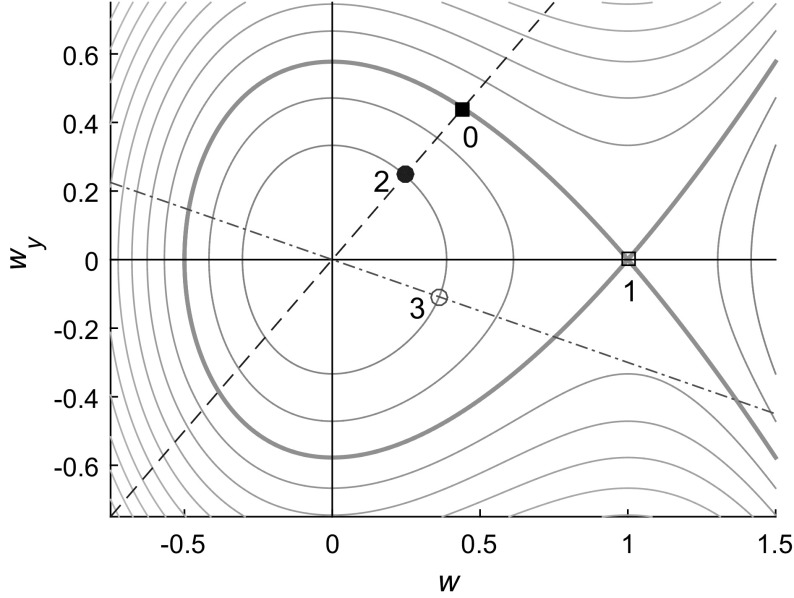
(Color figure online) Sketch of the phase portrait of the Hamiltonian ODEs (14) (*solid curves*). *Dashed* and *dash-dotted straight lines* indicate lines $z=\alpha w$ and $z=-\beta w$ on which solutions must begin (and for a finite domain, end), respectively, at $y=0$ and $y=L$. Points $(w_0,z_0), \ldots , (w_3,z_3)$ are indicated by the labels 0, 1, 2, 3

The 2D dynamical system (14) has two equilibrium points (0, 0) and (1, 0) which are a centre and a saddle point, respectively, and a sketch of the phase portrait is shown in Fig. 5.

The required boundary conditions are satisfied for the unique trajectory of (14) that satisfies $z=\alpha w$ at $y=0$ and $(w,z) \rightarrow (1,0)$ as $y \rightarrow \infty$ and for which both *w* and *z* are positive for all $y \ge 0$. These conditions hold for the unique trajectory that forms a section of the stable manifold of the saddle point at (1, 0), starting at the point in the $w>0$, $z>0$ quadrant where the line $z=\alpha w$ intersects this stable manifold, illustrated in Fig. 5 by the intersection at the point $(w_0,z_0)$ of the (blue) dashed line and the homoclinic orbit (thick curve) which forms the stable manifold of (1, 0). The homoclinic orbit has the analytic expression

17 $$\begin{aligned} w(y) = 1 - \frac{3}{2} \mathrm {sech}^2 \left( \frac{(y+y_0)}{2} \right) , \end{aligned}$$

for the transverse structure of the FKPP front, where $y_0$ is an as yet undetermined constant of integration. The form (17) clearly satisfies the condition $w \rightarrow 1$ as $y \rightarrow \infty$. Fixing the condition $w_y=\alpha w$ at $y=0$ enables us to determine $y_0$, implicitly, via

18 $$\begin{aligned} \frac{3}{2} \tanh \left( \frac{y_0}{2}\right) = \alpha \left[ \cosh ^2 \left( \frac{y_0}{2} \right) - \frac{3}{2} \right] . \end{aligned}$$

**Fig. 6:**
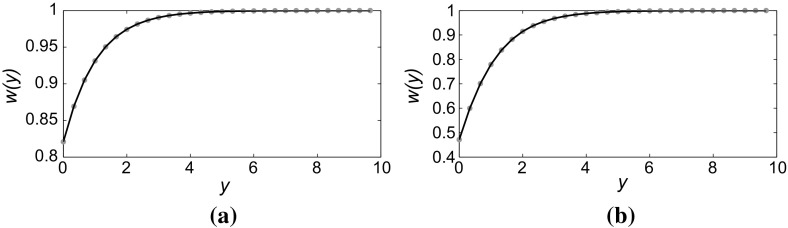
(Color figure online) Transverse structure (i.e. in *y*) of Fisher–KPP fronts propagating in the positive *x* direction in a strip $0 \le x \le 400$ and $0 \le y \le 10$. Plots of *w*(*y*) at fixed *x* far behind the propagating front, obtained from (17) where $y_0$ is determined as a function of $\alpha$ by solving (18) (*solid black line*) and numerical results (*blue dots*). **a** $\alpha =0.2$; **b** $\alpha =0.9$

Since the left-hand side of (18) increases monotonically from zero and asymptotically approaches 3 / 2 as $y_0$ increases to infinity, and the right-hand side increases monotonically from $-\alpha /2$ at $y_0=0$ to very large positive values as $y_0$ increases, it is clear that for any fixed $\alpha >0$ Eq. (18) has a unique positive solution $y_0$. Indeed in is clear by inspection of (18) that every such positive solution must satisfy $y_0 \ge 2 \mathrm {cosh}^{-1}\sqrt{3/2} > 0$. Figure 6 illustrates the form of the transverse structure of the front for two cases: $\alpha =0.2$ and $\alpha =0.9$. For the larger value of $\alpha$ the distortion is much more pronounced.

For the case of a strip $0 \le y \le L$ with either a no-flux or a partially absorbing boundary condition at $y=L$, the solution near $y=0$ is close to the solution for a half-plane. The analytic solution in this case can be constructed implicitly using the phase portrait shown in Fig. 5 since the finite width *L* now gives an additional constraint which selects a unique (part of a) trajectory $\gamma$ that must begin on the line $z=\alpha w$ (say at the point $(w_2,z_2=\alpha w_2)$) and end on the line $z=-\beta w$, (at the point, e.g. $(w_3,z_3=-\beta w_3)$) as illustrated in Fig. 5.

The finite width constraint can be expressed through the requirement that the contour containing the trajectory must correspond to a value of *H* that satisfies

19 $$\begin{aligned} L = \int _0^L \ dy = \int _{\gamma } \frac{\mathrm{d}w}{(2H(w_2,\alpha w_2) + 2w^3/3-w^2)^{1/2}}, \end{aligned}$$

which follows from the Hamiltonian (15), starting from the initial condition $(w_2,z_2)$ and setting $H=H(w_2,z_2)$ which is determined by the initial condition, and integrating along the trajectory $\gamma$ starting at the point $(w_2,z_2)$ indicated by the blue solid circle and ending at the open circle at $(w_3,z_3)$ contained in the level set $H=H(w_2,z_2)$, as illustrated in Fig. 5. For a specified finite value of *L*, the constraint (19) enables us to select a trajectory in the phase plane that satisfies both this constraint and the required boundary conditions, showing that a solution indeed exists for any positive *L*.

The trajectory in Fig. 5 starting at the black square at $(w_0,z_0)$ and ending at the saddle point at $(w_1,z_1)=(1,0)$ describes the solution for the half-space problem for which the solution at $y=0$ must satisfy $w_y=\alpha w$, and for which $w \rightarrow 1$ and $w_y \rightarrow 0$ as $y \rightarrow \infty$.

### Collapse Behind the Front

**Fig. 7:**
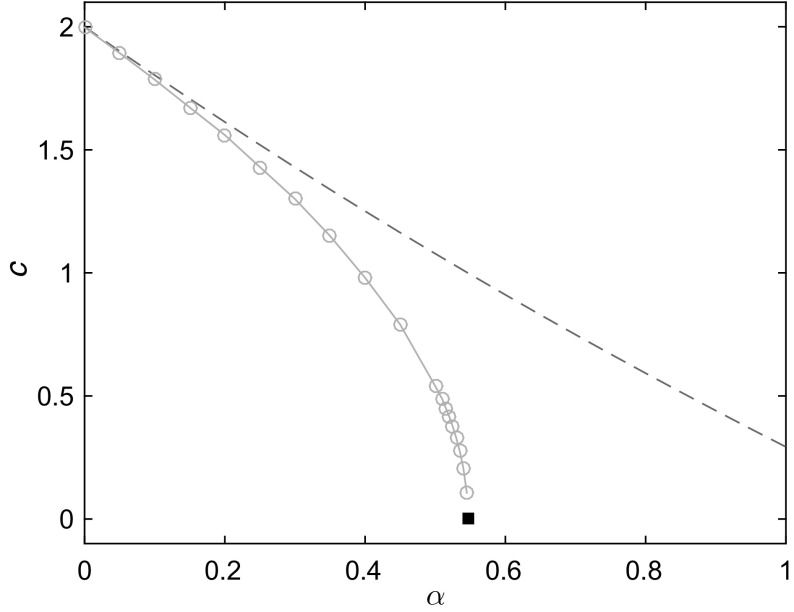
(Color figure online) Comparison of theoretical and numerically computed front propagation speeds *c* as a function of $\alpha$ (taking $\beta =0$) in a narrow domain of width $L=0.5$. *Blue dashed line* is the theoretical prediction of the speed from the leading edge calculation (6). *Red* symbols and *solid line* are numerically computed speeds. The speed of the front falls to zero at $\alpha \approx 0.546$. The *black square* indicates the prediction of this point, $\alpha =\tan (0.5)$ from (21)

A necessary condition for the propagation of stable travelling waves is that the solution behind the front discussed in Sect. 3.1 is also stable. Numerical investigations reveal that this may not be the case in very narrow domains. A consequence of this instability is that travelling waves can no longer exist. In fact, in very narrow domains the speed of propagation of travelling waves departs significantly from the theoretical value computed at the leading edge, as illustrated in Fig. 7. The wavespeed falls to zero at a point (indicated in Fig. 7 by the black square) at which the solution behind the front also fails to remain positive as the front travels forwards.

For this value of $\alpha$, the effect of the boundary condition becomes too significant for the strip to support a positive population behind the front. The critical point at which this occurs can be computed in a very similar way to the leading edge computation, since *u* is small so we can linearise, and also look for *x*-independent solutions, i.e. we investigate the linear stability of the solution $u=0$ to the linearised equation

20 $$\begin{aligned} u_t = u_{yy} + u, \end{aligned}$$

subject to the boundary conditions (2). Setting $u(y,t)=\mathrm {e}^{\sigma t}v(y)$ and defining $q:=\sqrt{1-\sigma }$ leads to an eigenfunction of the form

$$\begin{aligned} v(y)= A \cos q y + B \sin qy. \end{aligned}$$

After applying the boundary conditions at $y=0$ and $y=L$ we find that *q* must satisfy the condition

$$\begin{aligned} \alpha + \beta = \left( q - \frac{\alpha \beta }{q} \right) \tan qL. \end{aligned}$$

The critical case is where the growth rate $\sigma =0$, i.e. $q=1$ which implies the relation

$$\begin{aligned} \alpha + \beta = (1-\alpha \beta ) \tan L, \end{aligned}$$

for collapse to take place. For fixed $\beta$ the line in the $(L, \alpha )$ plane along which collapse takes place can be written as

21 $$\begin{aligned} \alpha = \frac{\tan L - \beta }{1+\beta \tan L}. \end{aligned}$$

**Fig. 8:**
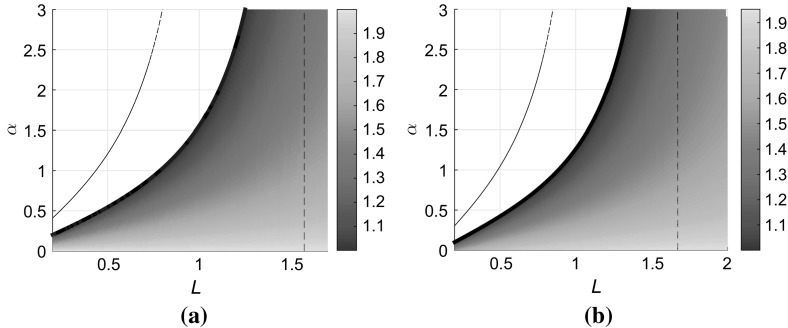
(Color figure online) Front propagation speed *c* in the $(L,\alpha )$ plane. **a** The case of a no-flux boundary at $y=L$; **b** the case of a partially absorbing boundary at $y=L$ with parameter $\beta =0.1$. In each plot, the coloured region indicates the existence of a stable travelling wave with speed given by the *colourbar*. The *solid line* at the boundary of the *coloured region* indicates the curve $L_0(\alpha )$ at which the front collapses; *dashed vertical lines* indicate the value of $L_c$. The *thin dash-dotted* line indicates the curve $L= L_m(\alpha )$ on which the propagation speed falls to zero according to the linear theory at the leading edge

It is instructive to compare the location of this line to the condition (10) deduced in Sect. 2 for the line on which the travelling wave speed is predicted to fall to zero, based on the calculation at the leading edge. A direct calculation shows that the inequality

$$\begin{aligned} \frac{\tan L - \beta }{1+\beta \tan L}< & {} \frac{2 \tan L\sqrt{2} - \beta \sqrt{2}}{\sqrt{2} + \beta \tan L\sqrt{2}}, \end{aligned}$$

holds for all positive values of $\beta$ and *L*, so that the collapse condition (21) always lies below the curve (10) in the $(L,\alpha )$ plane, as illustrated in Fig. 8. We conclude that the travelling wave ceases to exist due to the inability of the strip to sustain a positive population behind it, rather than because the invasion speed at the leading edge falls to zero.

## Discussion

In this paper we have analysed the widely used FKPP model of front propagation to look at 2D front propagation near a boundary that models a constant fractional loss of the local population density. This extension of the 1D FKPP model has received very little attention in the literature previously. We are motivated in particular by the experiments reported by Möbius et al. (2015) who observed a change in the shape of the propagating front near boundaries that could be modelled as ‘partially absorbing’ in the way we set out above.

Our results show that in wide strips, the speed of propagation depends heavily on the nature of the boundary conditions, but is still well approximated by the linearised regime ahead of the front. The wavespeed that is computed directly from the linearised problem in a bounded strip tends to overestimate that obtained from numerical simulations. In a half-space the wavespeed remains constant but the shape of the wave near the boundary depends on the boundary condition parameter. Explicitly we derive the expression $\tan \theta = 1/\alpha$ for the angle $\theta$ that contours of constant *u* make with the reactive boundary at which we impose the boundary condition $u_y=\alpha u$.

If the strip is sufficiently narrow, then when the influence of the boundary conditions is sufficiently strong, the travelling wave ceases to exist: it collapses at the point at which the regime behind the front can no longer sustain a positive population. Moreover, in narrow strips the wavespeed predicted by the linearised calculation at the leading edge ceases to be a good approximation to the speed of travelling waves computed numerically: the wavespeed is clearly determined as part of the fully nonlinear nature of the problem.

It is worth commenting briefly on the collapse phenomenon since the linear stability of the trivial state behind the front appears to be closely related to discussions in the literature of ‘critical patch sizes’ for populations in finite domains, as discussed by various authors including Skellam (1951), Kierstead and Slobodkin (1953), and more recently Ryabov and Blasius (2008). In particular the KiSS model (using the notation of these authors for a population density *P*(*x*, *t*))

$$\begin{aligned} P_t= & {} \mu _0 P + D P_{xx}, \qquad \text {on} \ 0 \le x \le L \\ P(0,t)= & {} P(L,t)=0, \end{aligned}$$

which these authors discuss is essentially the linearised FKPP equation with Dirichlet boundary conditions, i.e. the limiting version of (20) with reactive boundaries as $\alpha ,\beta \rightarrow \infty$. That the KiSS model is able to sustain positive population density *P*(*x*, *t*) only if $L \ge \pi \sqrt{D/\mu _0}$ is for exactly the same reasons as our model in the regime far behind the front. The nonlinear extension of the KiSS model, in the form

$$\begin{aligned} P_t= & {} \mu _0 P \left( 1-\frac{P}{K} \right) + D P_{xx}, \qquad \text {on} \ 0 \le x \le L \\ P(0,t)= & {} P(L,t)=0, \end{aligned}$$

with a logistic nonlinearity, has a stationary solution that can be expressed in terms of elliptic functions, and the critical patch size (i.e. domain length *L*) for persistence remains the same: we require $L \ge \pi \sqrt{D/\mu _0}$ for persistence.

Ludwig et al. (1979) considered an extension of the KiSS model in which the domain is the real line, but the mortality $\mu$ is negative outside a finite interval:

22 $$\begin{aligned} \mu (x) = \left\{ \begin{array}{rl} \mu _0 \quad &{} \text {for} \ 0< x < L \\ -m \quad &{} \text {for} \ x \le 0, \ \text {or} \ x \ge L. \end{array} \right. \end{aligned}$$

For this model, Ludwig et al. (1979) find that a population can persist if the domain size *L* satisfies

23 $$\begin{aligned} L \ge 2 \sqrt{\frac{D}{\mu _0}} \tan ^{-1} \sqrt{\frac{m}{\mu _0}}, \end{aligned}$$

which for positive *m* and $\mu _0$ implies that the population cannot exist in domains strictly smaller than $\sqrt{D/\mu _0}$. Even for small *m*, a critical domain size exists and can be approximated by $L \approx 2 \sqrt{Dm}/\mu _0$ when $0 < m \ll 1$.

It is interesting to compare these results with conditions (7) and (11) for the case of reactive boundaries considered here. Condition (23) implies that for every choice of the mortality *m* and the other problem parameters there exists a critical patch size, tending to zero as $m \rightarrow 0$, and $L \approx 2\sqrt{m}$ for small *m*, and tending to $\pi$ as $m \rightarrow \infty$. The same is true in the problem considered here: for any positive value of $\alpha$, there is a minimum domain size given by solving (7) below which the front will collapse and no travelling wave solution appears to be possible. Hence (21) implicitly yields a critical patch size in the sense of the KiSS problem, and the critical patch size depends on the value of the boundary parameters $\alpha$ and $\beta$: the front collapses if these boundary parameters are sufficiently large.

In terms of future directions there are three avenues that we intend to investigate in further work. The first is to compare these solutions for the Fisher–KPP PDE with the results of stochastic simulations for front propagation, incorporating the effects of the partially absorbing boundary at the microscopic level (Erban and Chapman 2007). The second direction is to consider further extensions of these kinds of boundary condition, for example introducing spatially inhomogeneous boundary conditions. The third direction is to vary the functional form of the PDE model (1), as has been done by many authors; either replacing the nonlinear term with a continuous but piecewise-affine construction, or considering more general nonlinearities. A further avenue is of course to make rigorous the statements that we conjecture here, and to provide the same level of proof for the 2D FKPP equation as is available for its 1D counterpart. We intend to report on these directions in future papers.
